# Associations of crying, sleeping, and feeding problems in early childhood and perceived social support with emotional disorders in adulthood

**DOI:** 10.1186/s12888-023-04854-1

**Published:** 2023-06-02

**Authors:** Julia Jaekel, Kati Heinonen, Nicole Baumann, Ayten Bilgin, Riikka Pyhälä, Christian Sorg, Katri Räikkönen, Dieter Wolke

**Affiliations:** 1 Psychology, University of Oulu, Oulu, Finland; 2 Department of Psychology, University of Warwick, Coventry, UK; 3 Department of Population Health Sciences, University of Leicester, Leicester, UK; 4 Department of Psychology and Logopedics, University of Helsinki, Helsinki, Finland; 5 Psychology/Welfare Sciences, Faculty of Social Sciences, Tampere University, Tampere, Finland; 6 Department of Psychology, University of Essex, Colchester, UK; 7 Department of Neuroradiology and Klinikum rechts der Isar, Technische Universität München, München, Germany; 8 Department of Psychiatry, Klinikum rechts der Isar, Technische Universität München, München, Germany; 9 TUM-NIC Neuroimaging Center Technische Universität München, München, Germany; 10 Warwick Medical School, University of Warwick, Coventry, UK; 11 Turner Institute for Brain and Mental Health, School of Psychology Sciences, Monash University, Melbourne, Australia

**Keywords:** Regulatory problems, Life-course, Clinical diagnoses, Anxiety disorder, Mood disorder, Social support, Protection

## Abstract

**Background:**

Multiple or persistent crying, sleeping, or feeding problems in early childhood (regulatory problems) are associated with increased internalizing symptoms in adulthood. Unknown is whether early regulatory problems are associated with emotional disorders in adulthood, and what psychosocial factors may provide protection. We tested whether early childhood multiple or persistent regulatory problems are associated with a higher risk of (a) any mood and anxiety disorder in adulthood; (b) perceiving no social support in adulthood; and (c) whether social support provides protection from mood and anxiety disorders among participants who had multiple/persistent regulatory problems and those who never had regulatory problems.

**Methods:**

Data from two prospective longitudinal studies in Germany (*n* = 297) and Finland (*n* = 342) was included (*N* = 639). Regulatory problems were assessed at 5, 20, and 56 months with the same standardized parental interviews and neurological examinations. In adulthood (24–30 years), emotional disorders were assessed with diagnostic interviews and social support with questionnaires.

**Results:**

Children with multiple/persistent regulatory problems (*n* = 132) had a higher risk of any mood disorder (odds ratio (OR) = 1.81 [95% confidence interval = 1.01–3.23]) and of not having any social support from peers and friends (OR = 1.67 [1.07–2.58]) in adulthood than children who never had regulatory problems. Social support from peers and friends provided protection from mood disorders, but only among adults who never had regulatory problems (OR = 4.03 [2.16–7.94]; *p* = .039 for regulatory problems x social support interaction).

**Conclusions:**

Children with multiple/persistent regulatory problems are at increased risk of mood disorders in young adulthood. Social support from peers and friends may, however, only provide protection from mood disorders in individuals who never had regulatory problems.

**Supplementary Information:**

The online version contains supplementary material available at 10.1186/s12888-023-04854-1.

## Background

The definition of infant regulatory problems (RPs) includes excessive crying beyond 3 months of age, and feeding and sleeping problems beyond 6 months of age [1]. About 20% of infants experience any of these RPs during their first year of life [2, 3], whereas 2–9% have several RPs concurrently (i.e., multiple RPs) and/or persistently across more than one assessment point during infancy and toddlerhood [1, 2, 4, 5]. Most early RPs are transient, however, the more severe combinations of multiple or persistent RPs have been associated with increased risk for long-term behaviour and mental health problems in childhood [2, 3, 6–10], adolescence [5], and adulthood [2, 11–13].

There is some evidence from prospective longitudinal studies of associations between early RPs and specific emotional problems, but diagnostic studies of clinical disorders are rare. For instance, it has been found that excessive infant crying is associated with an increased risk for mood problems at age 5–6 years, but not with general anxiety symptoms [14]. Parental report of severe sleeping difficulties at age 5 years has been associated with an increased risk of depression at 34 years [15], and multiple/persistent early RPs have been associated with internalizing problems and depressive symptoms in adulthood [11, 13].

Emotional disorders include both mood and anxiety disorders. While anxiety disorders may often have their age of onset in childhood, mood disorders typically have a later onset with prevalence rising rapidly from early adolescence into young adulthood [16, 17]. In the general population, the lifetime prevalence of mood and anxiety disorders between 18 and 29 years has been estimated at 21.4% and 30.2% [18], whereas the point prevalence in young adulthood averages at 10.5% and 12.3%, respectively [19]. However, we do not know whether children with multiple/persistent early RPs compared with children without early RPs are at an increased risk of any mood or any anxiety disorder in adulthood. This knowledge will be helpful for screening and health services planning.

In addition, it is critically important to identify variables that can protect young adults from emotional disorders. One factor which may influence the association between multiple/persistent RPs and the risk for emotional disorders in adulthood is social support. Humans are ultra-social creatures [20] and supportive social relationships are essential for our long-term health and quality of life [21]. For instance, previous studies have documented that social support from romantic partners can provide protection against the development of psychiatric problems [22, 23], and specifically of mood and anxiety disorders [24]. Likewise, social support from peers and friends is of critical importance for individuals struggling with emotional problems, possibly especially those with early RPs in childhood. However, given the documented tendency of individuals with early RPs for an avoidant personality profile [12] as well as possible difficulties with regulating their own emotions and behaviour, we hypothesize that their social relationships with peers, friends, and romantic partners may be negatively affected. According to our knowledge, this has never been investigated. Moreover, it is unknown whether potentially protective effects of social support from peers, friends, and romantic partners on emotional disorders are different among adults who had multiple/persistent RPs in contrast to those without any RPs.

In the current study, we use data from a bi-national cohort of two large prospective longitudinal samples in two countries (Germany and Finland) that assessed early RPs with identical measures and have harmonized clinical diagnosis data on mood and anxiety disorders as well as social support in adulthood. The aim is to test whether (a) multiple RPs at age 5 months and/or persistent RPs over three time points (at least one problem at ages 5, 20, and 56 months) are associated with a higher risk for any mood or any anxiety disorder in young adulthood, (b) multiple/persistent RPs are associated with a higher risk of perceiving no social support from peers, friends, and romantic partners, and (c) whether social support similarly provides protection from mood and anxiety disorders in individuals who had multiple/persistent RPs and those who never had any RPs. We formulated three hypotheses:

1. Children with multiple/persistent RPs compared with children who never had RPs have a higher risk for any mood and any anxiety disorders in adulthood.
2. Children with multiple/persistent RPs compared with children who never had RPs are at increased risk for perceiving no social support from peers, friends, and romantic partners in adulthood.
3. Social support provides protection from mood and anxiety disorders in individuals with early RPs and in individuals who never had RPs.

## Methods

Data were collected as part of the prospective Bavarian-Finnish Longitudinal Study (BFLS) [25], a geographically defined birth cohort of neonatal at-risk children born in Southern Bavaria, Germany (Bavarian Longitudinal Study, *N* = 7,505) and in the county of Uusimaa, Finland (Arvo Ylppö Longitudinal Study, *N* = 1,535) in 1985/86. In addition, 916 and 658 healthy infants born at term in the same hospitals were recruited as controls in Germany and Finland, respectively. Parents were approached within 48 h of the infant’s hospital admission and gave written informed consent to participate. Ethical approval for the studies was granted by the ethics committees of the University of Munich Children’s Hospital, the Bavarian Health Council (Landesärztekammer Bayern), the University Hospital Bonn, the Helsinki City Maternity Hospital, the Helsinki University Central Hospital, the Jorvi Hospital, and the Coordinating Ethics Committee of the Helsinki and Uusimaa Hospital District. All adult participants gave fully informed written consent.

**Fig. 1:**
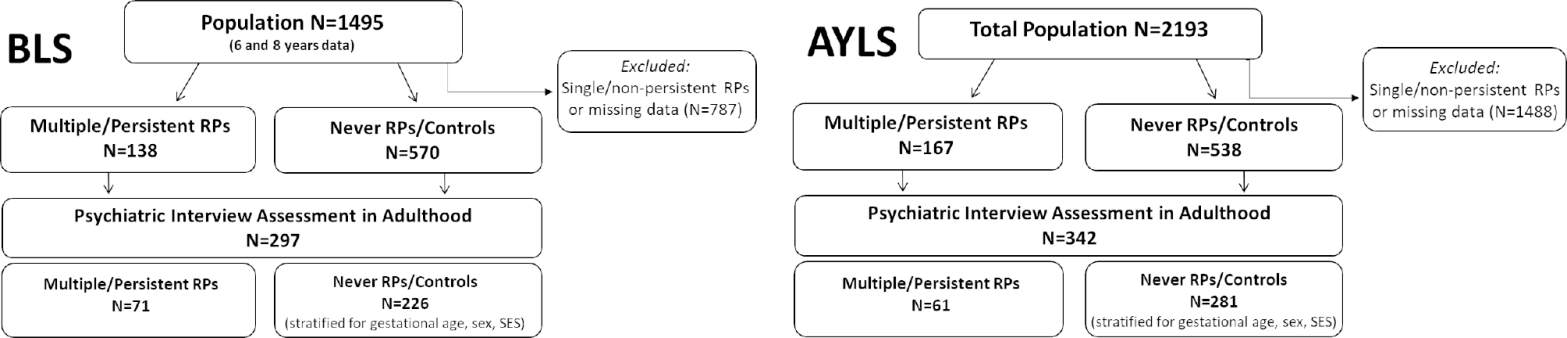
Sample flow diagram of the Bavarian-Finnish Longitudinal Study (BFLS) participants (total adult *N* = 639)

*Bavarian Longitudinal Study (BLS).* After the first phase of the study (birth to 56 months) the decision was made to reduce the sample size to *N* = 1,495 to allow for more intensive psychological and neurological assessments while preserving sufficient statistical power. Sampling criteria and dropout rates are provided elsewhere [26]. For this prospective case-control follow-up study in adulthood, we excluded (a) those participants who at any time between 5 and 56 months of age had a single or non-persistent RP (see Measures section for details), and (b) all cases with any missing data on crying, sleeping, or feeding problems between 5 and 56 months of age (*N* = 787, see Fig. 1). Of the remaining eligible 708 participants (*N* = 138 with multiple and/or persistent RPs and *N* = 570 without any RPs), we stratified and assessed *N* = 71 with multiple and/or persistent RPs and *N* = 226 without RPs with clinical psychiatric interviews in adulthood.

*Arvo Ylppö Longitudinal Study (AYLS).* Of the initial sample (*N* = 2,193), 2,086 participants were identified as alive and living in Finland in adulthood. For this prospective case-control study, 705 eligible participants were identified with complete data on multiple and/or persistent RPs (*N* = 167 with multiple and/or persistent RPs and *N* = 538 without any RPs) (Fig. 1). Of those, *N* = 61 participants with multiple and/or persistent RPs and *N* = 281 without RPs had complete clinical psychiatric interview data in adulthood.

As described above, all participants were assessed multiple times in childhood. For the design of the adult follow-up, we adopted a 2:1 sample design, with two control participants who never had any early childhood RPs per one adult participant who had had multiple and/or persistent RPs. The target sample size was determined by a-priori power analyses. In both cohorts, to ensure that the control participants were comparable to the group with multiple/persistent RPs, controls were selected and stratified according to sex, socioeconomic status (SES), and gestational age. Descriptive participant characteristics are shown in Table 1.

**Table 1:** Descriptive characteristics of the multiple/persistent RPs and never RP control participants (*N* = 639)

		Never RP, *n* = 507	Multiple/persist. RPs, *n* = 132	*Mean difference* *(95% CI)*	*p*
Gestational age (weeks)		37.67 (3.60)	37.69 (3.72)	-0.02 (-0.71–0.68)	0.967
Sex (% female)		52.9	49.2	0.55 ^a^	0.459
Age at adult assessment		26.31 (1.68)	26.90 (1.96)	-0.59 (-0.96 – (-0.23))	0.002
Family SES at birth	Low % Medium/High %	18.9 81.1	25.0 75.0	2.39 ^**a**^	0.122
Cohort membership	BLS % AYLS %	44.6 55.4	53.8 46.2	3.57 ^a^	0.059
% with no social support from peers and friends ^b^		20.7	30.0	5.94 ^a^	0.019
% with no social support from romantic partners ^b^		50.9	66.7	10.50 ^a^	0.001

Please note: Data are presented as mean (standard deviation) if not indicated otherwise;

^a^*χ²-*value; ^b^ binary-coded index score; RPs = regulatory problems

## Measures

*Multiple and/or persistent RPs from 5 to 56 months.* At 5 months of age, paediatricians asked parents about their infant’s crying, feeding, and sleeping problems via a standardized interview as part of a neurodevelopmental assessment. At 20 and 56 months, sleeping and eating problems were assessed via standardized parental interviews and neurological examinations of oral motor function. All assessments were conducted by paediatricians trained to achieve an inter-rater reliability > 90% and receiving three-monthly booster workshops. The assessments at 5 and 20 months were corrected for prematurity, the assessment at 56 months was carried out according to chronological age. The definitions for crying, feeding, and sleeping problems at 5 months and sleeping and eating problems at 20 and 56 months have been described previously [9]. Children diagnosed with multiple RPs had two or three RPs at 5 months (BLS: 13%, AYLS: 12%). Persistent RPs were defined as having at least one RP at 5, 20, and at 56 months of age (BLS: 14%, AYLS: 9%). Subsequently, multiple and/or persistent RPs were combined into one binary variable: 0 = never RPs, 1 = multiple/persistent RPs.

*Diagnosis of anxiety and mood disorders at 26 years.* Clinical Munich Composite International Diagnostic Interviews (M-CIDI [27, 28]) were conducted at 24–30 years. Assessors were qualified experienced psychologists (BLS) and expert-supervised master’s-level psychology students (AYLS), trained in the interview administration and blind to group membership [24, 29]. Anxiety and mood disorder diagnoses during a 12 month period were obtained according to DSM-IV criteria. Definitions of any anxiety or mood disorders included all DSM-IV defined subtype diagnoses. The M-CIDI has good concordance with the Structured Clinical Interview for DSM-IV (SCID) [30].

*Social support*. Information on non-biological social relationships (i.e., peers, friends, romantic partners) was obtained using questions from a standardized Life Course Interview and the Young Adult Self-Report [31] (administered in the BLS) or Adult Self Report [32] (administered in the AYLS), respectively. We extracted and harmonized critical items from these assessments that covered (a) social support from peers and friends, and (b) social support from romantic partners (Table S1) [24, 33]. These were summed and due to skewed item distribution recoded into two binary index scores for main analyses (0 = at least some support, 1 = no support). The two scores correlated with Spearman’s *rho* = 0.15, *p* < .001.

*Confounding variables*. Gestational age at birth was determined from maternal reports of the last menstrual period and serial ultrasounds during pregnancy. Information on family socioeconomic status (SES) at birth was obtained by standardized parental interviews within the first 10 days of life, including the occupation of the self-identified head of each family together with the highest educational qualification held by either parent. Composite indices were then coded into a binary score (1 = low, 0 = medium/high SES). We also included biological sex, age at assessment in adulthood, and cohort membership (BLS vs. AYLS) as confounding variables.

*Statistical analyses.* Data were analysed with SPSS 27. RP group comparisons of any anxiety and any mood disorder were carried out with binary logistic regressions. Interaction terms of RPs x social support in logistic regressions of any mood and any anxiety disorder tested if social support provided protection from these disorders similarly or differently in individuals with multiple/persistent and those who never had RPs. Analyses were adjusted for gestational age, biological sex, family SES, age at assessment in adulthood, and cohort membership (BLS vs. AYLS).

## Results

Table 1 shows that there were no significant differences between children with multiple/persistent RPs and those who never had RPs with regards to sex, gestational age, and family SES at birth. However, children who had multiple/persistent RPs were older at the time of the adulthood follow-up assessment compared to those who never had RPs (Table 1).

**Table 2:** Prevalence of DSM-IV emotional disorder diagnoses (%) comparing participants with multiple/persistent RPs and controls (*N* = 639)

DSM IV Diagnoses	Never RP *n* = 507	Multiple/persist. RPs *n* = 132	OR*	95% CI*	p*
**Any mood disorder**	60 (11.8%)	21 (15.9%)	1.81	(1.01–3.23)	0.046
**Any anxiety disorder**	95 (18.7%)	17 (12.9%)	0.76	(0.42–1.36)	0.354

* adjusted for gestational age, biological sex, and family SES at birth, age at assessment in adulthood, and cohort membership (BLS vs. AYLS); RPs = regulatory problems

Children with multiple/persistent RPs had significantly higher unadjusted rates and adjusted odds ratios of any mood disorder in adulthood than children who never had RPs (Table 2). The association with any anxiety disorders in adulthood was not significant (Table 2). These findings partially confirmed hypothesis 1.

Children with multiple/persistent RPs reported significantly higher unadjusted rates of no perceived social support from peers and friends and from romantic partners in adulthood than those who never had RPs (Table 1). When adjusted for confounders, the association with no perceived social support from peers and friends remained significant (odds ratio (*OR)* = 1.67, 95% confidence interval (*CI*)=[1.07 to 2.58]) but was rendered non-significant regarding perceived support from romantic partners (*OR* = 1.56 [0.99 to 2.43]). These findings partially confirmed hypothesis 2.

**Fig. 2:**
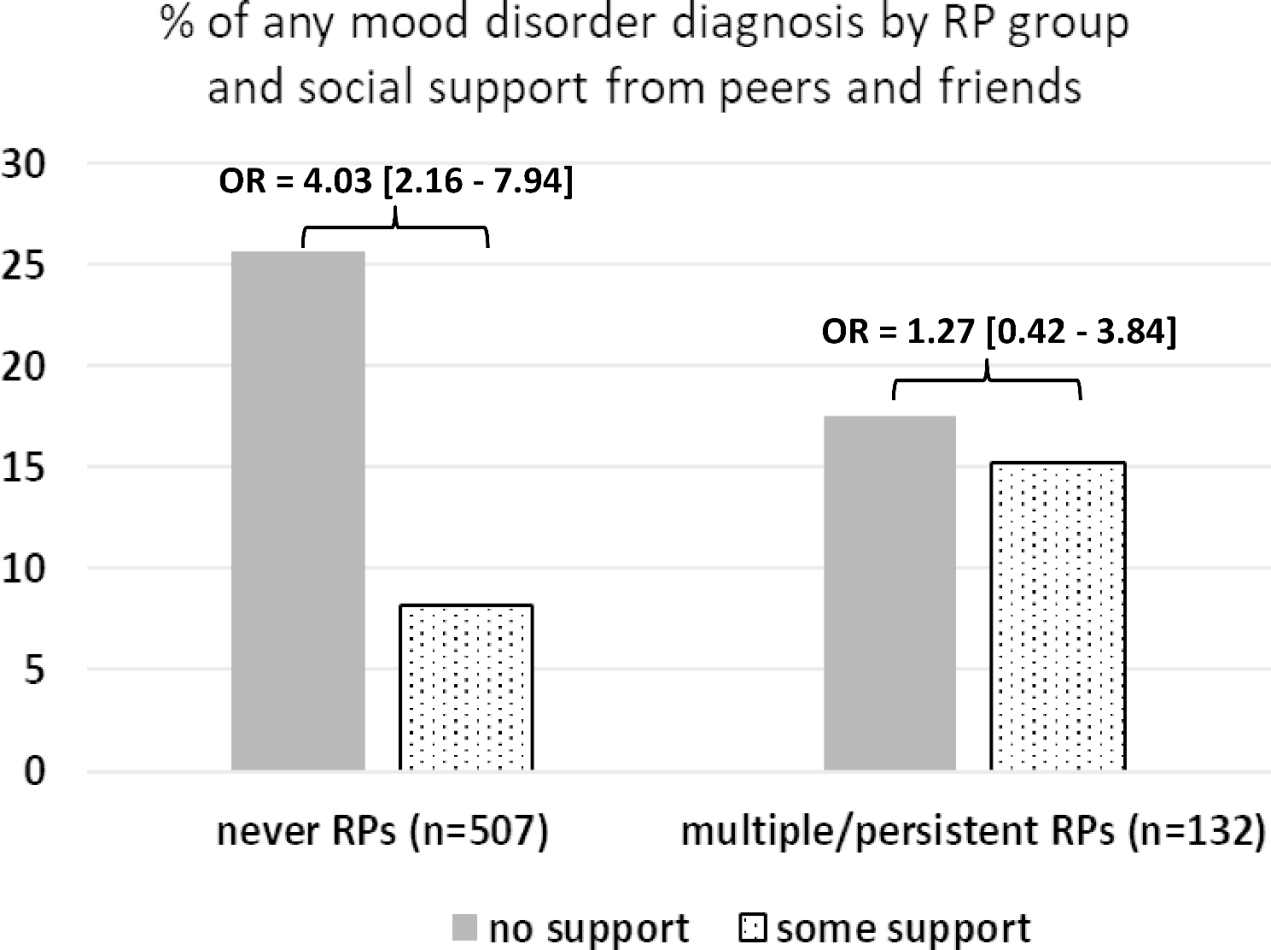
Unadjusted rates (%) and adjusted odds ratios (ORs) [95% confidence intervals] of any mood disorder diagnoses according to multiple/persistent regulatory problems (RPs) in early childhood and perceived social support from peers and friends in adulthood (*N* = 639)

To answer hypothesis 3, we first ran two logistic regression models with any mood and any anxiety disorder in adulthood as the two outcomes in a combined group of children with multiple/persistent and without any RPs, including both indices of social support from peers and friends and from romantic partners simultaneously. Adults who perceived having no social support from peers and friends compared with those who reported to have such support had higher adjusted ORs of any mood (*OR* = 2.68 [1.56 to 4.58]) and any anxiety disorder (*OR* = 2.37 [1.47 to 3.82]). In these models perceived social support from romantic partners was not significantly associated with any mood (*OR* = 1.51 [0.89 to 2.56]) or any anxiety disorder (*OR* = 1.16 [0.74 to 1.83]) and was therefore dropped from the subsequent models. We then examined whether the protective association of social support from peers and friends with any mood or any anxiety disorder in adulthood differed among children with multiple/persistent RPs versus those who never had RPs. Table 3 shows that the interaction effect of multiple/persistent RPs with social support was significant on any mood disorder as the outcome (0.27 [0.08–0.94], *p* = .039). Figure 2 displays this interaction and shows that in children who never had RPs, those who did not perceive having social support from peers and friends in adulthood had higher unadjusted rates and adjusted ORs (4.03 [2.16–7.94]) of any mood disorder than those who perceived having at least some support. In children with multiple/persistent RPs, the unadjusted rates and adjusted ORs of any mood disorder between those who perceived having and those who perceived having no social support were not different (Fig. 2). The interaction between multiple/persistent RPs and social support on any anxiety disorder was not significant (Table 3).

**Table 3:** Associations between emotional disorder diagnoses, multiple/persistent early RPs and social support from peers and friends (*N* = 639)

*Dependent variable*	Any mood disorder	Any anxiety disorder
	*OR (95% CI)*	*OR (95% CI)*
**Multiple/persist. RPs**	2.69 (1.31–5.51)**	0.79 (0.38–1.65)
**No social support from peers and friends**	3.93 (2.14–7.20)***	2.56 (1.52–4.32)***
**Interaction** (*multiple/persistent RPs x no social support from peers and friends*)	0.27 (0.08–0.94)*	0.74 (0.22–2.51)
Gestational age at birth	0.96 (0.90–1.02)	1.01 (0.95–1.07)
Biological sex	3.04 (1.75–5.27)***	2.16 (1.37–3.39)**
Low SES at birth	0.63 (0.33–1.21)	1.33 (0.79–2.23)
Age at adulthood assessment	0.54 (0.41–0.70)***	0.54 (0.42–0.68)***
Cohort membership	3.19 (1.69–6.01)***	2.29 (1.33–3.96)***

*= *p* < .05; **= *p* < .01; ***= *p* < .001; RPs = regulatory problems

## Discussion

This prospective study of two parallel cohorts from Finland and Germany found that children with multiple/persistent RPs compared with children who never had RPs were at increased risk of mood disorders but not anxiety disorders in adulthood. In addition, children with multiple/persistent RPs compared with children who never had RPs were at increased risk of having no perceived social support from peers and friends in adulthood. Among children who never had RPs, those who reported to not have any social support from peers and friends were at higher risk for any mood disorder in adulthood than those who perceived to have at least some support. This protective pattern did not apply to individuals with multiple/persistent early RPs.

In accordance with the documented increased risk for life-course trajectories of dysregulation and mental health symptoms among children with early RPs [2, 5, 11], our results add that they are also at an increased risk for clinical diagnoses of mood disorders. In contrast, our nonsignificant finding of risk for anxiety disorders is somewhat reassuring, especially in light of the substantial comorbidity between mood and anxiety disorder diagnoses [34], which highlights a specific association of early multiple or persistent RPs on mood disorders. The current study is the first of this kind and one can only broadly speculate about possible underlying mechanisms and individual differences such as social experiences, personal attribution styles, neurodevelopmental functioning, HPA axis dysregulation, or epigenetic programming. Moreover, there may also be differences in genetic liability with regard to risk for RPs, and we cannot rule out associations with the genetic liability that underpins mood disorders. Future replication studies in other samples of individuals who had RPs in childhood are warranted.

Children with multiple or persistent RPs were more likely to not have social support from peers and friends in adulthood than those who never had RPs. There are several possible explanations for why early RPs may cascade into lack of support from social relationships in adults. Early RPs may disrupt socio-emotional synchrony and quality of parent-infant interaction, which provide the foundation for later social interactions with peers, friends, and romantic partners [35–37]. In addition, early RPs have been associated with reduced intrinsic functional connectivity of the brain’s default mode network, which is involved in a wide range of functions and particularly relevant for the interactions of the individual with their social environment [12]. The development of social behaviour is characterized by continuous bidirectional feedback loops over time [38, 39]. For instance, caregivers as the earliest interactive partners may respond in certain ways to episodes of dysregulated behaviour, and children with RPs may incorporate these responses into their own expectations of co-regulation through behavioural exchange as they grow up. There are however important differences between biological (e.g., parents) and non-biological (e.g., friends) social relationships. Peers and friends, for instance, may respond very differently to episodes of dysregulated behaviour than mothers and fathers, possibly by withdrawing from the relationship. One could speculate that repeatedly unmet expectations of behavioural exchanges and co-regulation with interactive partners may cascade into social relationship problems and perceived lack of social support later in life. Indeed, our findings suggest that children with multiple/persistent early RPs do not only perceive that they have less social support than their peers in adulthood, but also that their mood disorder risk is not affected by social support from friends and peers. According to well-established theories of risk and resilience, protective effects among the at-risk group in this study would have been somewhat expected [40]. Nevertheless, our data did not confirm it, and this is exactly why empirical testing of theories and objective interpretation of study results is critically important to our disciplines. Moreover, the finding is in direct contrast to one of the leading theories in developmental psychology, which proposes increased differential susceptibility among individuals with early difficult temperaments [41]. We have documented previously that theory-driven expectations of resilience and differential susceptibility among individuals who had early regulatory problems are not always applicable in adulthood [11]. We can only speculate why social support from friends and peers does not decrease the mood disorder risk among young adults who had multiple/persistent RPs in their early childhood, especially when considering that RPs were associated with more frequent reports of no social support. Perhaps their previous social experiences have not helped them overcome dysregulated moods, which could be in line with neurological models of learned helplessness and social defeat [42]. Perhaps the substantial normative changes adolescent brains undergo, which are associated with psychiatric disorders [43], are qualitatively different among individuals who previously had multiple/persistent RPs, which would be in line with novel pubertal recalibration models [44]. Replication studies are warranted.

Nevertheless, the finding of children who never had RPs being at lower risk for any mood disorders in adulthood if they perceived having social support from friends and peers provides practically important and novel evidence of preventative mechanisms in the general population. While social relationships may provide a resource of wellbeing more effectively for some people than for others, the vast majority of individuals very likely benefits from social support [45]. Therefore, a screening for lack of social support from friends and peers can offer very valuable pointers as to who should be screened and be eligible for interventions that can protect from mood disorders. A previous study from Germany has shown that perceived support from romantic partners may play an important role with regard to a lower risk for any mood disorders in young adulthood [24], but this finding was not replicated in the current sample from Finland and Germany. This could be due to slight cultural differences or small methodological variations, or due to the competitive modelling approach, where support from romantic partners did not independently explain significant variation in outcomes on top of the variation explained by social support from peers and friends. Future observational and intervention studies should consider assessing and evaluating both, social support from peers and friends as well as the support received from romantic partners.

The current study has several strengths. It is the first longitudinal investigation of anxiety and mood disorders in adulthood using clinical diagnoses in a bi-national cohort study including two large prospective samples of individuals who had multiple/persistent RPs in early childhood compared with a matched group of children who never had RPs. This cross-cultural design offers some generalisability of findings beyond just one country, however, it is still limited to Western, educated, industrialised, rich, and democratic (WEIRD) populations [46]. Our data includes repeated prospective assessments of RPs in infancy and toddlerhood, made via both clinical parent interviews and neurological examinations, and clinical diagnostic interviews in adulthood in both countries. We utilized gold-standard diagnostic interviews and our reported prevalence rates are in accordance with what has been reported in general population studies before. Specifically, the prevalence rates for any mood and anxiety disorder diagnoses reported here are in line with expectations based on other normal population samples from studies with similar designs including data from Europe [19]. This is of interest, as meta-analyses show that prevalence rates vary widely by world region [47]. All analyses were adjusted for a range of confounders.

There are also limitations. Maternal anxiety and mood problems have been associated with the development of infant regulatory problems [48]. However, early regulatory problems may also be precursors of maternal depression rather than the result of it [49]. Nevertheless, this question would need to be tested within a gene-environment study design, while genetic information was not included in this study and gene-environment correlations or interactions could not be investigated [50]. Other limitations include the lack of assessing other contributing factors to individuals’ risk for mood disorders in adulthood, such as experiencing bullying and victimisation, substance use, or low sensitive parenting behaviour. These factors were not investigated in conjunction with RPs as part of the current study. Moreover, RPs were not assessed via structured diaries. This was not feasible in these two prospective cohorts due to the often-reported high attrition rates in diary studies. Moreover, the samples of both cohorts included participants who were born preterm (i.e., born before 37 weeks of gestation), however, we controlled for the influence of gestational age in all analyses. Some readers may speculate about the numbers in Table 1 - these are showing distributions according to sample size based on recruitment criteria. Specifically, these do not show a higher risk among BLS children than among AYLS children to be diagnosed with multiple/persistent RPs, but merely a slightly different (non-significant) percentage of the BLS compared with AYLS adult participants who were selected for psychiatric assessment in adulthood who had multiple/persistent RPs. Irrespective of their diagnostic prevalence, mood and anxiety problems occur in the population across a symptomatic continuum rather than a categorical diagnosis. Here, our variables of any mood and anxiety diagnoses did not allow detection of subclinical symptom levels, although these may significantly affect young adults’ wellbeing and daily functioning [51]. Finally, social support was assessed in adulthood, thus causality as a protective factor for mood disorders cannot be inferred.

## Conclusions

This prospective longitudinal study shows that children with multiple/persistent RPs are at higher risk of any mood disorder and of not having social support in adulthood than their peers who never had early RPs; there is no heightened risk for any anxiety disorder. Social support from peers and friends is associated with a lower risk of any mood and any anxiety disorder, but the lower risk of mood disorders is seen only among individuals who never had early RPs. Accordingly, for children with multiple/persistent RPs, perceived social support from peers and friends in adulthood does not have a protective effect against mood disorders.

## Electronic supplementary material

Below is the link to the electronic supplementary material.

Supplementary Material 1

## Data Availability

The datasets analysed during the current study are not publicly available due to protection of participant privacy rights but deidentified summary data are available from the corresponding author on reasonable request.

## List of abbreviations

Regulatory problems: RPs
odds ratio: OR
confidence interval: CI
Bavarian-Finnish Longitudinal Study: BFLS
Bavarian Longitudinal Study: BLS
Arvo Ylppö Longitudinal Study: AYLS
socioeconomic status: SES
Clinical Munich Composite International Diagnostic Interview: M-CIDI
Structured Clinical Interview for DSM-IV: SCID
