# Supplementary material for: Associations of crying, sleeping, and feeding problems in early childhood and perceived social support with emotional disorders in adulthood
Source: BMC Psychiatry. 2023 Jun 2;23:394. doi: 10.1186/s12888-023-04854-1 (PMC10239120; doi:10.1186/s12888-023-04854-1)
Supplement: Supplementary file 1 — Supplementary Material 1 [file 12888_2023_4854_MOESM1_ESM.docx]

**Table S1. Item distributions for the social support from peers and friends and social support from romantic partner index scores**

| **No social support from peers and friends** | **Never RP  (*n* = 507)** | **Multiple/persist. RPs (*n* = 132)** |
| --- | --- | --- |
| Not liked by others | 12.23 % | 20.45 % |
| Trouble making or keeping friends | 15.38 % | 29.55 % |
| No best friend | 10.85 % | 20.45 % |
| No circle of friends | 44.38 % | 45.45 % |
| **No social support from romantic partner** |  |  |
| Never had sexual intercourse | 5.72 % | 9.09 % |
| Never in serious romantic relationship | 20.32 % | 25.76 % |
| Frequent conflicts with partner | 14.20 % | 18.18 % |
| Poor relations with the opposite sex | 12.23 % | 12.12 % |

Please note: All items were summarized and coded into binary scores (0=at least some support, 1=no support)
